# Supplementary figures and images for: Ambient Temperature and the Frequency of Subsequent Heart Failure Decompensations in an Emergency Department
Source: Biomedicines. 2025 Apr 27;13(5):1054. doi: 10.3390/biomedicines13051054 (PMC12109250; doi:10.3390/biomedicines13051054)

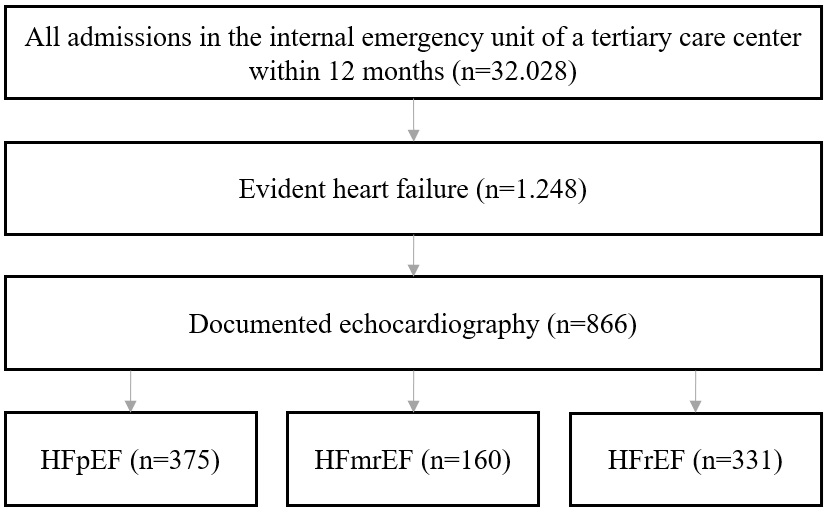

Supplement: Supplementary file 1 [file biomedicines-13-01054-s001.zip › biomedicines-3571182-supplementary.jpg]
